# Fate and functional roles of Prominin 1^+^ cells in liver injury and cancer

**DOI:** 10.1038/s41598-020-76458-8

**Published:** 2020-11-10

**Authors:** Raymond Wu, Stephanie Pan, Yibu Chen, Yasuhiro Nakano, Meng Li, Steven Balog, Hidekazu Tsukamoto

**Affiliations:** 1 Southern California Research Center for ALPD and Cirrhosis and Department of Pathology, Keck School of Medicine, University of Southern California, Los Angeles, CA USA; 2 USC Libraries Bioinformatics Services, University of Southern California, Los Angeles, CA USA; 3 Center for Matrix Biology and Medicine, Graduate School of Medicine, Tokai University, Isehara, Japan; 4 Greater Los Angeles VA Healthcare System, Los Angeles, CA USA; 5 Present Address: Department of Developmental and Regenerative Biology, Medical Research Institute, Tokyo Medical and Dental University, Tokyo, Japan

**Keywords:** Alcoholic liver disease, Liver cancer, Cell biology, Computational biology and bioinformatics, Molecular biology, Stem cells, Gastroenterology, Medical research, Oncology

## Abstract

Prominin 1 (PROM1) is one of a few clinically relevant progenitor markers in human alcoholic hepatitis (AH) and hepatocellular carcinoma (HCC), and mouse liver tumor initiating stem cell-like cells (TICs). However, the origin, fate and functions of PROM1^+^ cells in AH and HCC are unknown. Here we show by genetic lineage tracing that PROM1^+^ cells are derived in part from hepatocytes in AH and become tumor cells in mice with diethyl nitrosamine (DEN)-initiated, Western alcohol diet-promoted liver tumorigenesis. Our RNA sequencing analysis of mouse PROM1^+^ cells, reveals transcriptomic landscapes indicative of their identities as ductular reaction progenitors (DRPs) and TICs. Indeed, single-cell RNA sequencing reveals two subpopulations of *Prom1*^+^ *Afp*^–^ DRPs and *Prom1*^+^ *Afp*^+^ TICs in the DEN-WAD model. Integrated bioinformatic analysis identifies Discodin Domain Receptor 1 (*DDR1*) as a uniquely upregulated and patient-relevant gene in PROM1^+^ cells in AH and HCC. Translational relevance of *DDR1* is supported by its marked elevation in HCC which is inversely associated with patient survival. Further, knockdown of *Ddr1* suppresses the growth of TICs and TIC-derived tumor growth in mice. These results suggest the importance of PROM1^+^ cells in the evolution of liver cancer and DDR1 as a potential driver of this process.

## Introduction

Liver progenitor cells (LPCs) proliferate after massive parenchymal loss or/and suppressed hepatocyte proliferation in different liver diseases^1^. However, the origin and fate of these cells in liver injury and cancer development are unclear^2^. LPCs are identified by immunophenotype markers such as A6, SOX9, KRT19 (CK19), EpCAM and PROM1 and generated from biliary epithelial cells or hepatocytes through hepatocyte-to-ductal metaplasia^3^. Tumor-initiating stem cell-like cells (TICs) also express the overlapping progenitor genes and uniquely possess self-renewing and tumor-initiating properties due to intrinsic TLR4-Nanog oncogenic pathway^4^. Therefore, LPCs and TICs may share the same or similar lineage, but a definite relationship between them is currently unknown.

PROM1 is a well-known marker of TICs in many cancers and used to define cancer stemness and disease severity in patients^5,6^. PROM1-positive (PROM1^+^) cells also accumulate in mouse liver injury models and livers of alcoholic hepatitis (AH) patients in the pattern commonly characterized as ductular reaction progenitors (DRPs). The expression of *PROM1* is also inversely associated with survival of AH patients^7^. These clinical findings underscore the potential importance of PROM1^+^ cells in chronic liver disease and cancer. A study using mice bearing *Prom1*^*C-L*^ which expresses Cre recombinase and *LacZ* genes in the *Prom1* locus, reports that PROM1^+^ cells are generated by proliferating ductular epithelial cells in response to chronic thioacetamide liver damage^8^. Another study using the same genetic mice, reports that PROM1^+^ cells from neonatal liver have high regenerative capacity, which is almost undetectable in the adult liver^9^. However, these PROM1^+^ cells reappear in adult mice upon feeding a diet containing 3,5-diethoxycarbonyl-1,4-dihydrocollidine (DDC), and such livers express similar stemness transcriptomes as those in neonatal liver. Further, PROM1^+^ cells are transformed to tumor cells and give rise to more aggressive liver tumors upon DDC feeding when they are forced to express oncogenes or mutant tumor suppressors^9^. Although HCC cells are not derived from cytokeratin 19 (*Krt19*) or osteopontin (*Spp1*) expressing biliary epithelial cells, they express higher levels of *Prom1* compared to non-tumor tissues, suggesting the contribution of hepatocyte-derived PROM1^+^ cells in HCC formation^10^.

Alcoholic liver disease (ALD) is one of the significant health-related financial burdens in the world^11^. AH represents the most severe spectrum of ALD with high mortality^12^ and is presented as acute syndrome superimposed on chronic liver disease characterized by intense polymorphonuclear cell (PMN) inflammation, the appearance of DRPs and jaundice. One study suggests that these DRPs in AH patients reflect cholangiocyte proliferation^13^. But it is largely unknown how DRPs emerge in AH or if and how they contribute to AH pathogenesis. To address these questions in an animal model, we developed a mouse model which reproduces histologic AH with DRP proliferation^14^. The model also reproduces clinical features of AH including portal hypertension as judged by splenomegaly, hypoalbuminemia, and increased plasma bilirubin. More importantly, an expansion of PROM1^+^ cells expressing SPP1, NANOG, and AFP is detected in the model^14^.

Understanding the mechanisms underlying how PROM1^+^ cells emerge and expand in AH mouse liver may reveal the potential pathogenic role of these cells in AH and liver cancer susceptibility. Unfortunately, epidemiology of liver cancer incidence in AH patients is lacking due primarily to the high mortality rate of AH patients and difficulties in long-term tracking and assessment of the patients after hospital discharge. However, heavy alcohol intake and Western diet are two top-ranked risk factors for both AH and liver cancer^15^. In the present study, we aimed to determine the origin and molecular characteristics of PROM1^+^ cells in AH by genetic lineage tracing and RNA sequencing (RNAseq) analysis and to assess the fate of PROM1^+^ cells in liver tumor development in mice given Western alcohol diet (WAD) by genetic tracing and single-cell RNA sequencing (scRNAseq).

## Results

### Transcriptome of PROM1^+^ cells from AH resembles known DRP and HCC gene signatures

**Figure 1:**
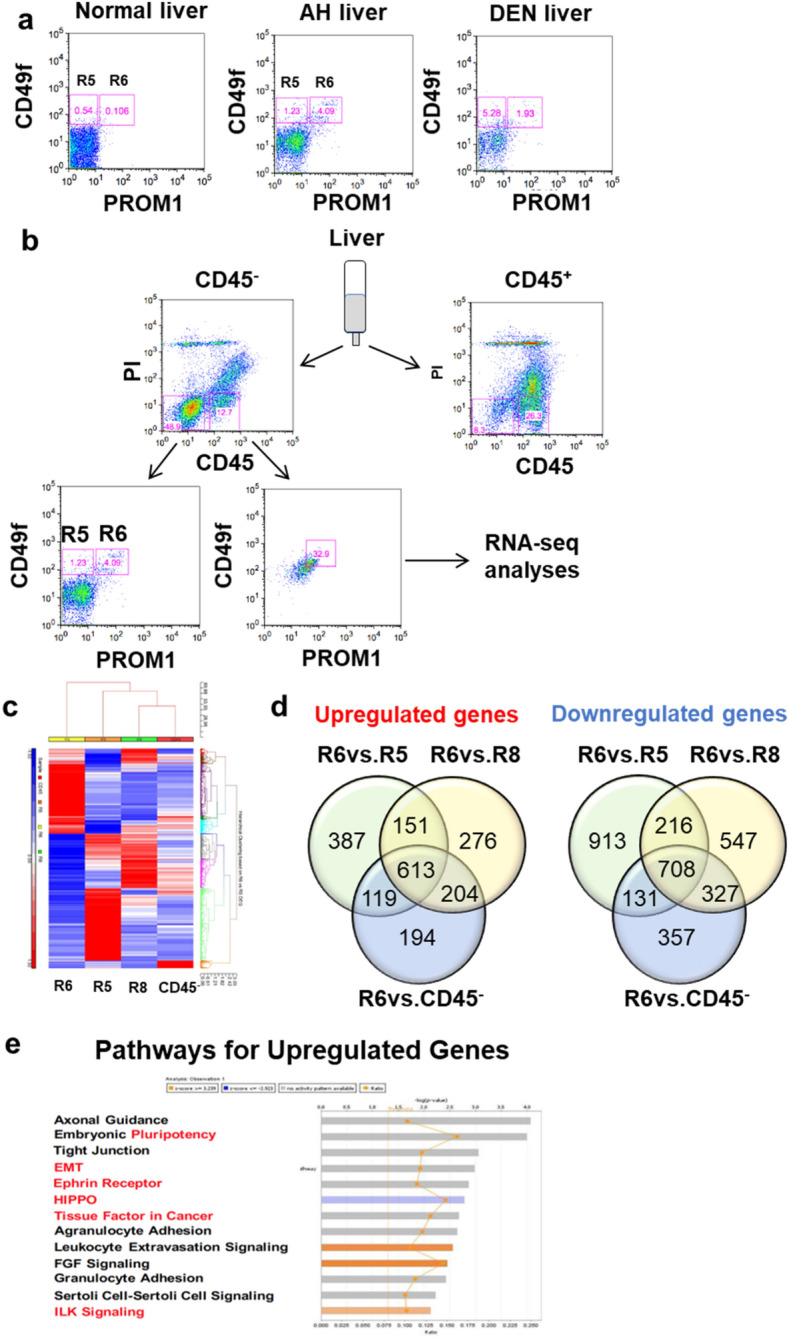
Progenitor and tumorigenic characteristic of PROM1^+^CD49f^+^ cells isolated from mouse model of alcoholic hepatitis (AH). (**a**) FACS plots of PROM1^+^CD4f^+^ cells isolated from normal mice, AH mice and DEN-initiated liver tumor mice. For DEN mice, 2-week old C57/BL6 mice were injected with 25 mg/kg of DEN intraperitoneally and fed with WAD for 5 months. (**b**) The flow chart shows how PROM1^+^CD49f^+^CD45^–^ cells were isolated from mouse liver. (**c**) Hierarchical clustering of PROM1^+^CD49f^+^CD45^–^ (R6), PROM1^–^CD49f^+^CD45^–^ (R5), PROM1^+^CD49f^+^CD45^+^ (R8) cells and CD45^–^ liver cells, is shown using a differentially expressed gene (DEG) set specific for R6 versus R5. (**d**) Venn diagrams show the number of upregulated and downregulated genes specific for PROM1^+^ liver cells (CD45^–^). (**e**) Ingenuity pathway analysis of DEG specific for PROM1^+^ liver cells, reveals known stemness and tumorigenic pathways highlighted in red, are significantly regulated.

To characterize a gene expression profile of PROM1^+^ cells in AH, we isolated PROM1^+^ cells from AH mouse livers^14^ by FACS using antibodies against PROM1 and CD49f which were used as stem cell markers for mouse liver TICs^4,16^. In normal mouse liver, a PROM1^+^CD49f^+^CD45^–^ population accounts for ~ 0.1% which increases to 1.93% in diethyl nitrosamine (DEN)-liver cancer model^4^. The yield of PROM1^+^CD49f^+^CD45^–^ cells from AH mouse livers ranged from 1 to 4% of sorted cells (Fig. 1a), suggesting PROM1^+^ cells are increased in the liver of AH mice. To characterize PROM1^+^ cells, PROM1^+^CD49f^+^CD45^–^ (**R6**) versus PROM1^–^ CD49f^+^CD45^–^ (**R5**) cells were sorted and total RNA was sequenced as shown in a flow chart in Fig. 1b. We also sorted PROM1^+^CD49f^+^ cells present in CD45^+^ population as hematopoietic PROM1^+^ cells (**R8**).

Total RNAseq analysis revealed a unique transcriptomic landscape of R6 PROM1^+^ cells as compared to R5 PROM1^–^ or R8 hematopoietic PROM1^+^ cells (Fig. 1c). To identify differentially regulated genes in R6 (PROM1^+^) cells, we compared gene expression profiles of R6 versus R5 (PROM1^–^), R6 versus R8 (hematopoietic PROM1^+^), and R6 versus all CD45^–^ sorted cells. As shown in the Venn diagrams in Fig. 1d, this analysis identified 613 upregulated and 708 downregulated genes specific for PROM1^+^ liver cells (Suppl. Table 3 in Excel format). The upregulated gene list included twofold or higher expression of genes considered as markers for progenitors (*Prom1*, *EpCAM*, *Sox9*), biliary epithelial cells (*Krt19*, *Hnf1β*, *Spp1*) and mature hepatocytes (*HNF4α*, *Alb*), suggesting hepatic PROM1^+^ cells in AH are bipotent progenitor cells. Ingenuity Pathway Analysis of the PROM1^+^ cell transcriptome, also revealed significantly regulated stemness and tumorigenesis pathways such as pluripotency, EMT, tissue factor in cancer, Ephrin receptor, HIPPO pathway, and ILK signaling, suggesting their potential involvement in liver tumorigenesis (Fig. 1e).

### Lineage of PROM1^+^ cells in AH and their fate in liver cancer

To determine how PROM1^+^ cells are generated in AH, we performed lineage tracing in the AH mouse model. To test the contribution of hepatocytes to the genesis of PROM1^+^ cells, we utilized two approaches to label hepatocytes in *Rosa26mTmG* reporter mice before AH induction: the use of the conventional *Albumin-Cre* (*Alb-Cre*) and the more mature hepatocyte specific *Transthyretin-Cre* (*Ttr-Cre*). *Alb-Cre*;* Rosa26mTmG* mice showed strong GFP expression by hepatocytes. Using these cells, we were able to establish the gating for GFP positive cells.

To generate *Ttr-Cre* mice, we injected AAV8-Ttr-Cre virus to *Rosa26mTmG* mice via tail vein and allowed 1 month for recombination to occur. The labeling efficiency was 98.8% for *Alb-Cre* mice and 99.2% for *Ttr-Cre* mice according to GFP positivity (Suppl. Figure 1b and 1c). To trace GFP^+^ hepatocytes to PROM1^+^ DRPs in the AH mice, we isolated CD45^–^ nonparenchymal liver cells by a low speed centrifugation to remove hepatocytes and magnetic depletion of CD45^+^ cells. The cells from C57/BL6 mice after the AH protocol were used as a negative control because the Western diet regimen contributed to high background fluorescence (Suppl. Figure 1d). The cells were sorted from *Alb-Cre;Rosa26mTmG* mice (Suppl. Figure 1g) and *Rosa26mTmG* mice injected with AAV8-Ttr-Cre (Suppl. Figure 1j) for PROM1 and GFP. From the cells from AH liver of *Alb-Cre;Rosa26mTmG* mice, PROM1^+^ /GFP^+^ cells were detected at 0.118% of frequency versus PROM1^+^ /GFP^–^ cells at 0.172%, suggesting 40.7% of PROM1^+^ cells are GFP-traced cells from *Alb-Cre*-based labeling in this representative mouse (Suppl. Figure 1g). Using *Ttr-Cre* labeling method which is considered more specific for hepatocytes^17^, the frequency of GFP^+^ /PROM1^+^ cells was shown to be 0.195% as compared to 0.691% of total PROM1^+^ cells with or without GFP expression, indicating 28.2% of the PROM1^+^ cells are hepatocyte-derived in this particular *Ttr-Cre;Rosa26mTmG* mouse subjected to AH (Suppl. Figure 1j, right panel). After repeating the same analysis in 4 AH mice, the average percentage of GFP^+^ /PROM1^+^ cells was determined to be 24% (Suppl. Figure 1k). These results suggest that PROM1^+^ cells are generated in part from mature hepatocytes in AH and the origin appears heterogeneous. The latter notion is also consistent with the recent finding of the heterogeneity of PROM1^+^ cells in HCC^18^.

**Figure 2:**
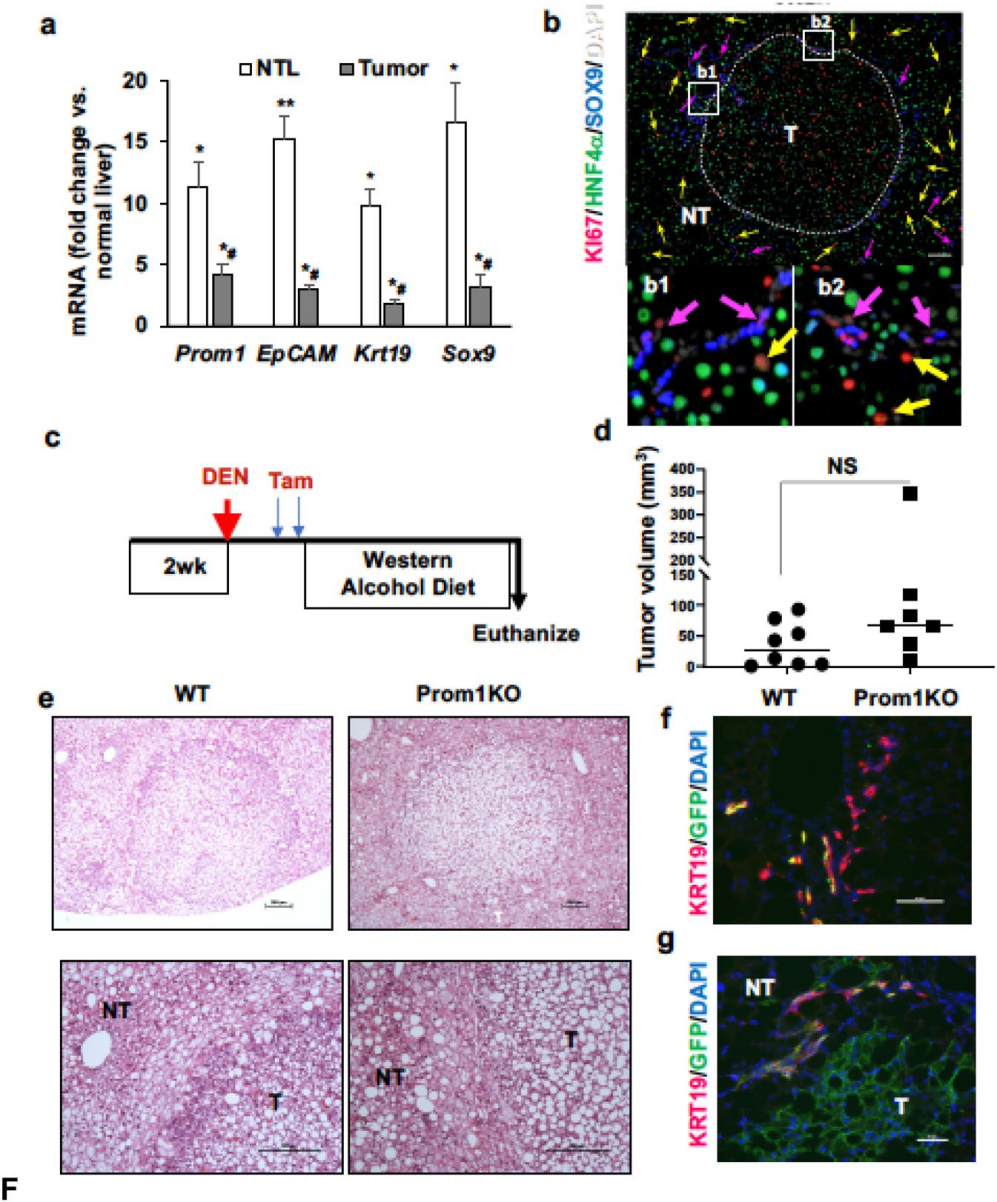
Lineage tracing of PROM1^+^ cells in a mouse model of DEN-initiated, WAD-promoted liver cancer. (**a**) Expression of DRP markers in non-tumor liver (NTL) versus tumor tissues. *p < 0.05 and **p < 0.01 compared to normal livers; #*p* < 0.05 compared to NTL by non-parametric student’s t-test (n = 6). (**b**) Representative co-immunofluorescent staining for hepatocyte marker (HNF4α), proliferation marker (Ki67) and a DRP marker (SOX9) of a tumor-bearing liver section from a DEN-WAD mouse with insets of enlarged views (**b1** and **b2**). Yellow and pink arrows indicate Ki67^+^HNF4α^+^ hepatocytes and Ki67^+^SOX9^+^ DRPs, respectively. Scale bar = 100 μm. (**c**) Schematic diagram of lineage tracing of PROM1^+^ cells using *Prom1*^*C-L*^*;Rosa26mTmG* mice in DEN-WAD model. Two-week old *Prom1*^*C-L*^*/Rosa26mTmG* mice with *Prom1*^*C-L*^ homozygosity (*Prom1KO*) and its wildtype counterparts were injected with 25 mg/kg DEN intraperitoneally. The mice were then injected with two doses of Tam 3 days apart before WAD was introduced. The mice were euthanized after 5 months of WAD feeding. (**d**) Total tumor volumes from *Prom1KO* and its wildtype were shown. Width (W) and length (L) of each visible tumor was measured with a caliper. Tumor volume was calculated with the following formula: (LxW^2^)/2. Statistical comparison was done with student’s t-test. (**e**) Representative tumor images of hematoxylin and eosin sections of WT and *Prom1KO* DEN-WAD mouse livers. The bottom images are magnifications of the top images with tumor (T) and nontumor (NT) areas. Scale bar = 200 μm. (**f**) Immunofluorescent (IF) staining of *Prom1*^*C-L*^*;Rosa26mTmG* liver sections showing KRT19^+^ (pink) DRP cells with some with co-localized GFP staining (yellow). Scale bar = 50 μm. (**g**) IF section from the same mouse shown in f, depicting GFP (green)-labeled tumor cells (T) and KRT19^+^ (pink) DRP cells in NTL area. Scale bar = 50 μm.

A subpopulation of PROM1^+^ cells observed in HCC is suggested to possess tumor initiating properties^4^. Indeed, the DRP markers such as *Prom1*, *EpCAM*, *Krt19*, and *Sox9* detected in AH, were also induced in non-tumor liver tissue (NTL) of mice subjected to DEN injection and tumor promotion with WAD as compared to normal liver or tumor tissues, suggesting a more selective increase of DRPs in NTL (Fig. 2a). Indeed, immunofluorescent microscopy detected proliferating DRPs were concentrated in NTL around the peripheral border of the tumor mass as Ki67^+^ SOX9^+^ cells (purple arrows, Fig. 2b), revealing a close spatial association of DRPs with tumor cells.

To determine the fate and role of PROM1^+^ cells in liver tumorigenesis, we traced PROM1^+^ cells using the DEN-initiated and WAD-promoted liver tumor model in *Prom1*^*C-L*^ mice, a strain engineered to have *CreERT2* and *Lacz* genes in the *Prom1* locus^19^. While this mouse serves to express CreERT2 under the *Prom1* promoter for labeling of PROM1^+^ cells, the homozygous strain of *Prom1*^*C-L/C-L*^ can also be used as *Prom1* knockout because it completely ablates the *Prom1* expression due to the biallelic knock-in of the CreERT2-Lacz cassette. We crossed *Prom1*^*C-L/C-L*^ mice with *Rosa26mTmG* mice to generate *Prom1 *^*C-L*^; *Rosa26mTmG* (*Prom1WT*) and *Prom1*^*C-L/C-L*^*;Rosa26mTmG* (*Prom1KO*) mice, injected DEN at 2-week age and fed WAD from 5-week age for 6 months. Tamoxifen (Tam) was injected to label PROM1^+^ cells at 3 weeks after DEN injection (Fig. 2c). Liver tumor development in *Prom1WT* and *Prom1KO* mice was assessed via gross and histologic examination (Fig. 2d,e). As predicted, *Prom1* expression in tumors of *Prom1*^*C-L/C-L*^*;Rosa26mTmG* (*Prom1KO*) mice were almost undetectable by qPCR analysis, validating their knockout status (Suppl. Figure 2a).

Surprisingly, total liver tumor volumes calculated in these two genetic groups, were not different (Fig. 2d), indicating PROM1 is not essential in liver tumorigenesis. To determine the fate of PROM1^+^ cells in tumor development, we stained the liver sections with visible tumors with anti-GFP and anti-KRT19 antibodies (Fig. 2f,g). While DRPs were detected as GFP^+^ KRT19^+^ cells in NTL region, tumor cells were also labeled with GFP but not for KRT19. These results suggest that PROM1^+^ cells give rise to liver tumor cells even though PROM1 is not functionally required for tumor development.

### scRNAseq analysis reveals Prom1^+^ TIC and DRP subpopulations in liver tumor

**Figure 3:**
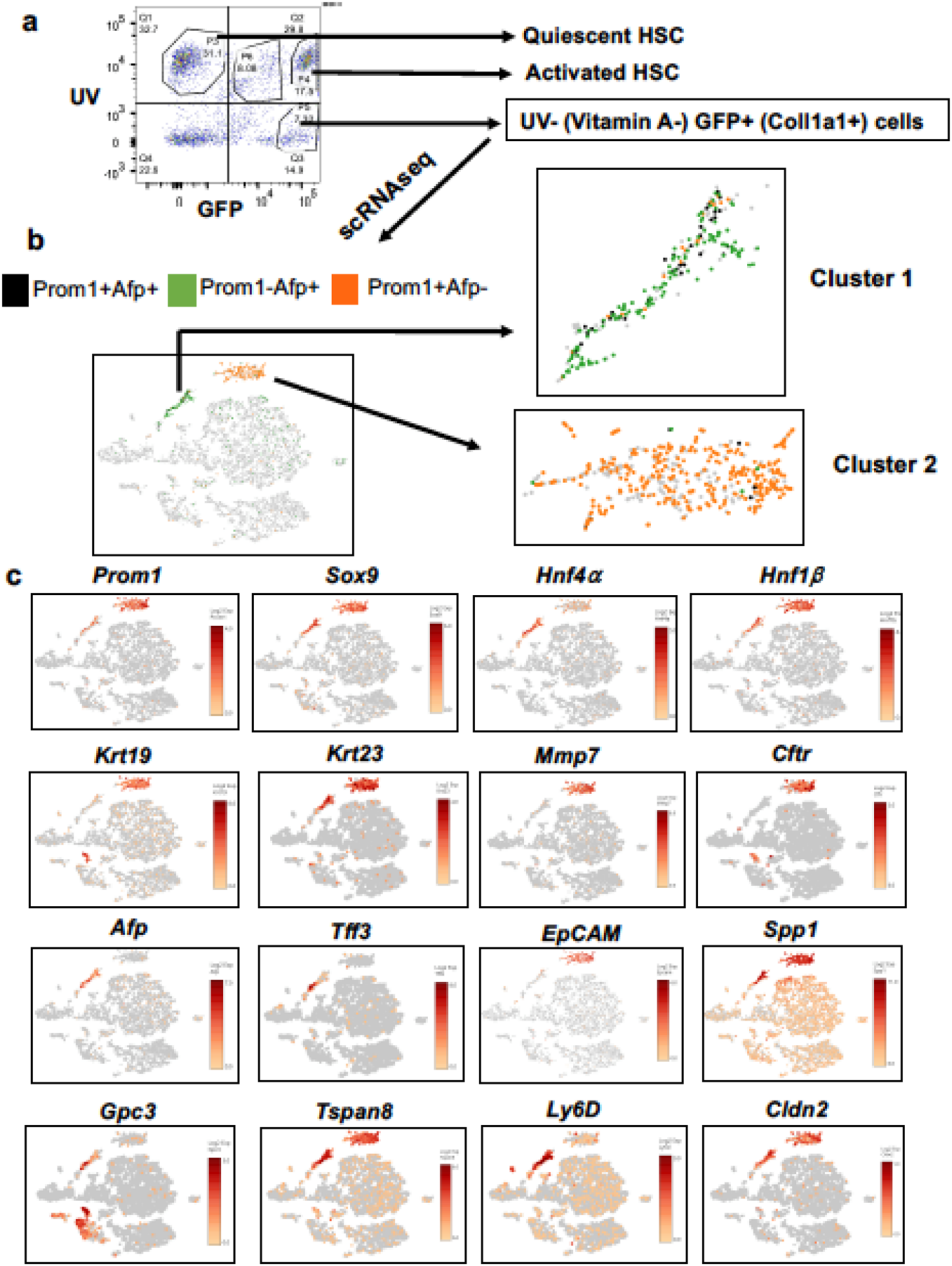
Identification of *Prom1*^+^ TICs and *Prom1*^+^ DRPs subpopulations from DEN mouse liver by scRNAseq. (**a**) The FACS plot depicting the sorting of UV^–^ GFP^+^ cells from DEN-WAD mouse liver cells. Non-parenchymal cells were isolated by collagenase perfusion from *Col1a1Cre;Rosa26mTmG* mice subjected the DEN-WAD regimen. The cells were sorted by FACS to remove UV^+^ hepatic stellate cells and to collect UV^–^GFP^+^ cells as *Col1a1* expressing and vitamin A negative cells. The scRNAseq library was prepared immediately after sorting. (**b**) The analysis detected *Prom1*^+^*Afp*^+^, *Prom1*^+^*Afp*^*–*^ and *Prom1*^*–*^*Afp*^+^ populations. The plots were created in the t-SNE format using filters of *Prom1* and *Afp* transcripts. (**c**) Co-expression analysis of *Prom1*^+^ cells with known markers of DRPs, TICs, tumor. The plots were created in the t-SNE format with gene/feature expression in Loupe browser. The scale bar represents log2-transformed normalized expression.

To understand the molecular characteristics of *Prom1*^+^ cells in the single cell resolution, we performed scRNAseq analysis. To enrich the LPCs and TICs, we have developed a unique approach of sorting *Col1a1*-expressing cells by FACS. The rationale for this approach is based on: (1) LPCs were previously reported to express high levels of *Col1a1* expression^3^; (2) our own results confirmed 23-fold and 90-fold higher expression of *Col1a1* in LPC line (PIL4) and TICs as compared to mouse primary hepatocytes, respectively (Suppl. Figure 3a); and (3) our additional data validated higher expression of *Col1a1* in PROM1^+^ versus PROM1^–^ cells isolated via MACS from the DEN-WAD mouse livers (Suppl. Figure 3b). We used *Col1a1*-driven Cre recombinase expression to label LPCs and TICs in *Col1a1Cre;Rosa26mTmG* mice subjected to the DEN-WAD protocol. Tumor bearing livers were perfused and digested with collagenase to collect liver cells. Mature hepatocytes were removed by low speed centrifugation, and the remaining liver cells were subjected to FACS sorting based on ultraviolet (UV) and GFP. UV-excited gating was used to exclude vitamin A-storing hepatic stellate cells (HSC), the major *Col1a1*-expressing cell type in the liver. The UV^–^GFP^+^ population was sorted as tumor-associated *Col1a1*-expressing cells and submitted for scRNAseq analysis (Fig. 3a). Barcode rank plot of the scRNAseq sample showed an excellent separation between the cell-associated barcodes and barcodes associated with empty partitions (Suppl. Figure 4). From a single cell library with the target cell number of 10,000, 8578 cells passed this quality control assessment. By this scRNAseq analysis, we indeed identified two subpopulations which included *Prom1*^+^ cells (Cluster 1 and 2) (Fig. 3b) with no or minimal expression of the HSC marker *Lrat*, portal fibroblast marker *Thy-1*, hematopoietic marker *Cd45*, and endothelial cell markers *Cd31* and *Cdh5* (Suppl. Figure 5a). As predicted, Cluster 1 and 2 cells expressed the biliary or/and progenitor markers such as *Sox9*, *Spp1*, *Hnf1b*, *Krt19*, *Krt23, Epcam* (Fig. 3c). To further validate these results, we assessed co-expression of these genes with *Prom1* at the single cell level. As shown in Suppl Fig. 5b, Cluster 1 and 2 are enriched with cells which co-expressed *Prom1* and *Sox9*, *Spp1*, *Hnf1b*, or *Krt23* and most *Prom1*^+^ cells in both clusters did not co-express the HSC, portal fibroblast, hematopoietic, and endothelial cell markers, validating these *Prom1*^+^ Cluster 1 and 2 cells are LPCs. The cells co-expressing *Prom1* and *Afp*, the known marker of liver tumor cells, were detected primarily in Cluster 1 (Suppl Fig. 5a and Fig. 3a). In fact, Cluster 1 contained both *Prom1*^+^ *Afp*^+^ and *Prom1*^–^*Afp*^+^ cells, suggesting TICs and tumor cells were enriched in this Cluster. *Prom1*^+^ *Afp*^–^ cells enriched in Cluster 2 are DRPs as the biliary/DRP markers such as *Hnf1b, Krt19*, *Epcam, Mmp7* and *Cftr* were more selectively expressed in Cluster 2 cells (Fig. 3c).

**Figure 4:**
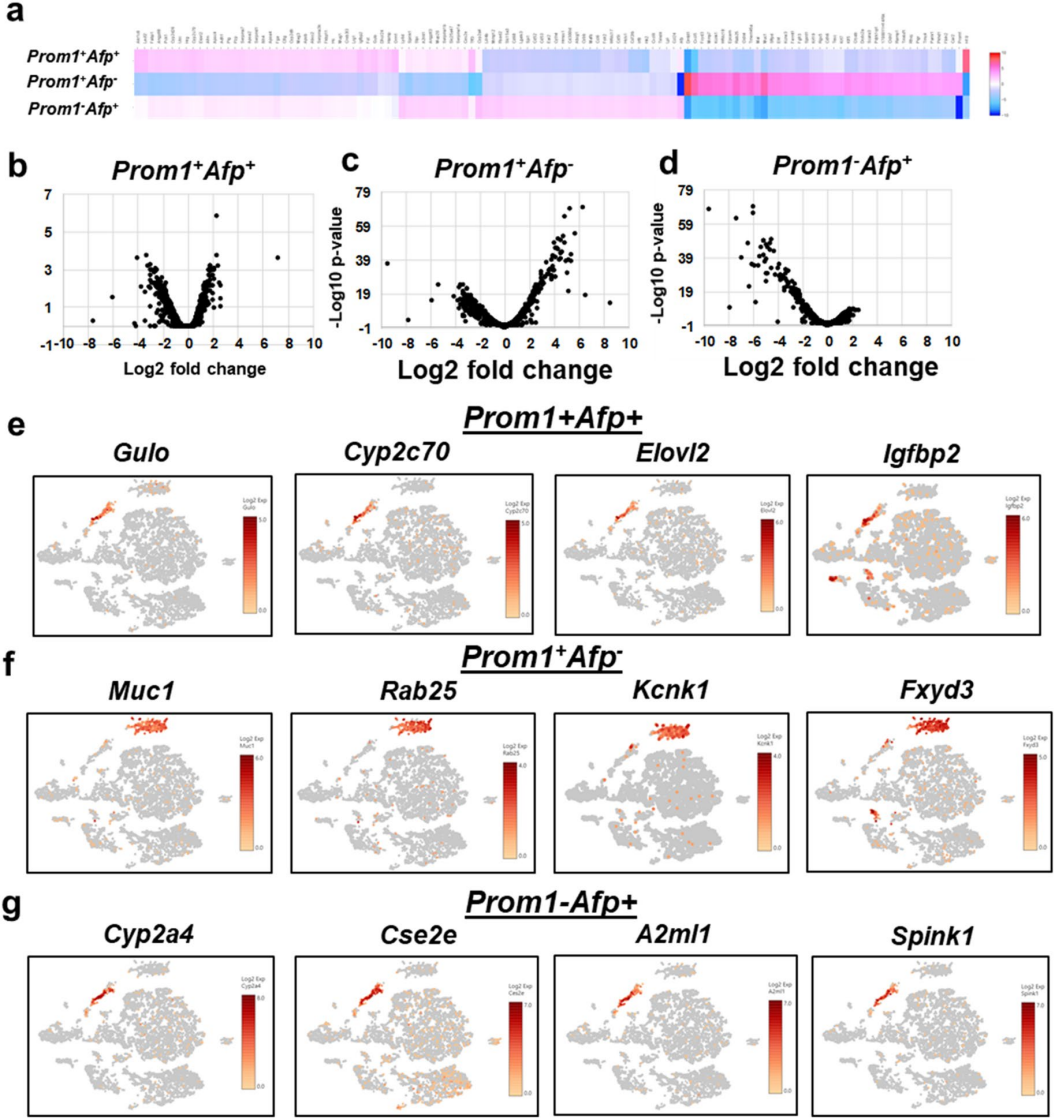
Novel transcript profiles specific for *Prom1*^+^*Afp*^+^, *Prom1*^+^*Afp*^*–*^ and *Prom1*^*–*^*Afp*^+^ populations. (**a**) The graph-based clustering of DEG sets for *Prom1*^+^*Afp*^+^, *Prom1*^+^*Afp*^*–*^ and *Prom1*^*–*^*Afp*^+^ cells. (**b**–**d**) volcano plots of transcript profiles from the three subpopulations. (**e**–**g**) Top representative novel transcripts identified by scRNAseq for each subpopulation are shown in t-SNE format as generated by the Loupe browser. The scale bar represents log2 transformed normalized expression.

**Figure 5:**
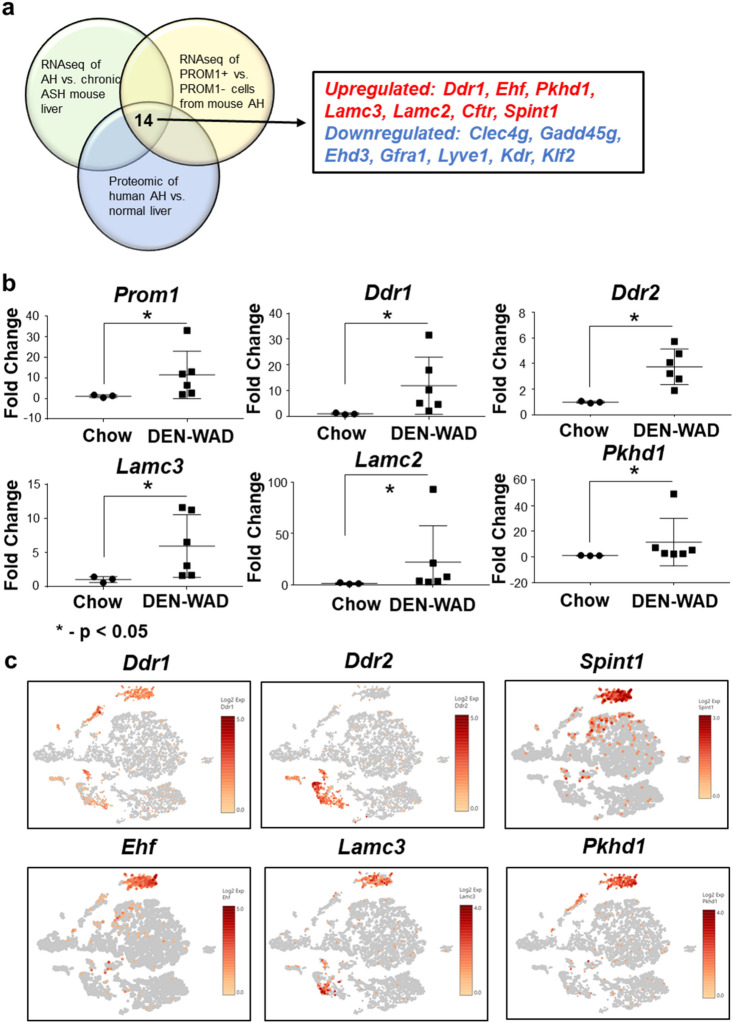
Unique upregulation of Discoidin Domain Deceptor 1 (*Ddr1*) in PROM1^+^ cells. (**a**) Integrated analysis of a RNAseq profile of PROM1^+^ cells from mouse AH, a RNAseq profile of the liver of mouse AH, and a proteomic profile of human AH versus control liver, reveals 7 upregulated and 7 downregulated genes common in all three comparisons. (**b**) Quantitative PCR analysis of DEN-WAD mouse livers versus normal livers, reveals that some of the upregulated genes shown by the integrated bioinformatic analysis are also induced in the liver of the mouse liver tumor model including *Ddr1*. *p < 0.05 based on non-parametric student’s t-test. (**c**) Expression of *Ddr1*, *Ddr2*, *Spint1*, *Ehf*, *Lamc3* and *Pkhd1* at the single cell level are shown in the t-SNE format generated by Loupe browser. The scale bar represents log2 transformed normalized expression.

We next assessed whether the known TIC/HCC transcripts are expressed in the scRNAseq profiles of the two clusters. For this, we assessed a set of genes reported to characterize cancer progenitor cells with tumor-initiating properties which were isolated from DEN-induced tumor bearing livers^20^. Many of the genes upregulated in these tumor initiating cells were expressed in our Cluster 1 cells including *Tff3*, *Tspan8*, *Gpc3*, *Ly6d*, *Cldn2* (Fig. 3c). These genes are involved in malignancies and cancer stemness^21–23^, further supporting the notion that Cluster 1 cells are TICs and tumor cells. We then acquired gene expression profiles unique to each of the three populations of *Prom1*^+^ *Afp*^+^ , *Prom1*^+^ *Afp*^–^, and *Prom1*^–^*Afp*^+^ cells by comparing among them by the locally distinguishing comparison feature of the Loupe Browser as depicted by heatmaps and volcano plots (Fig. 4a–d). As shown in the heatmaps, *Prom1*^+^ *Afp*^+^ and *Prom1*^–^*Afp*^+^ cells share coordinately upregulated genes most likely reflecting their common tumorigenic function yet with some discordant gene expression which may reflect genetic differences between TICs with stemness property and tumor cells which have lost the stemness. In contrast, *Prom1*^+^ *Afp*^–^ cells which we consider as DRPs have a clearly distinct gene expression pattern from other two Afp^+^ cell populations. Expression of 4 top genes uniquely expressed in each population are shown in Fig. 4e–g, and complete lists of the genes are provided in Suppl. Tables 4–6 in Excel format.

### Discoidin domain receptor 1 is a potential oncogenic driver of PROM1^+^ cells

To address the relevance of the transcriptomic data from PROM1^+^ cells to AH livers in mice and patients, we compared our PROM1^+^ cell RNAseq profile with a RNAseq profile of AH mouse liver and a previously reported proteomic profile of human AH liver^24^. This integrated analysis identified 7 upregulated and 7 downregulated genes that are common in PROM1^+^ cells, mouse and human AH livers (Fig. 5a). Since our results showed PROM1^+^ cells gave rise to liver tumor cells, we looked for potential oncogenic drivers unique to PROM1^+^ cells by first screening the expression of the 7 upregulated genes in NTL tissues of DEN/WAD mice compared to normal liver. This analysis revealed that *Prom1*, *Ddr1*, *Ddr2*, *Lamc2*, *Lamc3* and *Pkhd1* were induced significantly in DEN-WAD livers (Fig. 5b). Next, we examined the expression of these genes in cell clusters revealed by our scRNAseq analysis. Of these genes examined, *Ddr1* and *Pkhd1* expressions were detected in Cluster 1 and 2 (Fig. 5c). DDR1 is known as a receptor for collagen^25^ and implicated in oncogenesis, metastasis and chemoresistance of cancers of pancreas, lung, prostate, ovary, and colon^26–29^. *Ddr2, in contrast,* is expressed in clusters of activate *Lrat*^+^ HSC and *Thy1*^+^ portal fibroblasts (Fig. 5c and Suppl. Figure 5). *Spint1* and *Ehf* were expressed more selectively in DRPs in Cluster 2 (Fig. 5c), suggesting their importance in the biology of *Prom1*^+^ DRPs.

**Figure 6:**
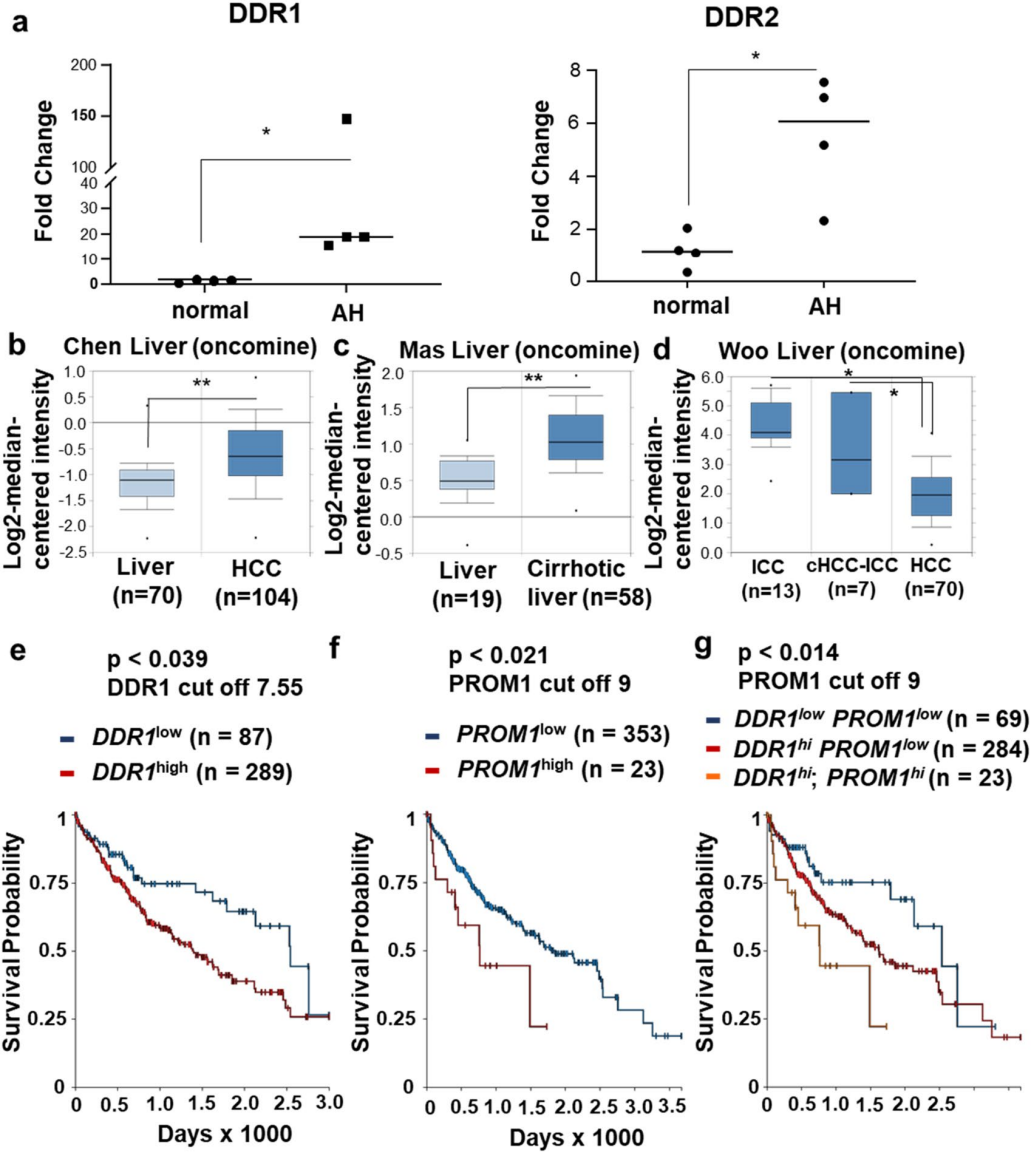
*DDR1* upregulation in liver cancer associated with poor patient survival. (**a**) *DDR1* and *DDR2* expression levels were measured by qPCR in normal human livers versus alcoholic hepatitis (AH) livers. *p < 0.05 based on non-parametric student’s t-test. (**b**,**c**) *DDR1* expression in HCC^30^ and cirrhotic^31^ human livers were compared to normal livers using the existing microarray data from Oncomine database^49^. (**d**) *DDR1* expression was compared in intrahepatic cholangiocarcinoma (ICC), combined HCC and ICC (HCC-ICC) and HCC in patients^30^. *p < 0.05 and **p < 0.01 based on the Oncomine database. (**e**–**g**) Kaplan–Meier analysis of TCGA HCC cohort data using UCSC Xena^32^, reveals an inverse relationship between the expression of *DDR1, PROM1* or both with patient survival. *p* values were calculated by log-rank test.

To confirm *DDR1* upregulation in AH in patients in our hands, we analyzed *DDR1* mRNA levels in liver tissues of AH patients versus healthy subjects received from the explant repository program of John Hopkins University. Indeed, this analysis revealed at least 20-fold higher *DDR1* expression in AH livers compared to the normal liver (Fig. 6a). *DDR2* level was also statistically higher in AH tissues than normal liver, but the fold induction was much lower than *DDR1*. To address the role of *DDR1* in human liver cancer, we performed the data mining approach. This bioinformatic analysis showed that *DDR1* expression was significantly elevated in both HCC^30^ (Fig. 6b) and cirrhotic livers^31^ (Fig. 6c) compared to normal livers.

Patients with combined HCC-intrahepatic cholangiocarcinoma (ICC) have poor prognosis^33^. ICC is characterized by higher expression of liver progenitor markers such as *EpCAM* and *KRT19*, suggestive of expanded TICs and/or DRPs. Indeed, *DDR1* expression is higher in patients with ICC or combined HCC-ICC compared to those with HCC alone (Fig. 6d)^34^.

Next we assessed the relationship of *DDR1* and *PROM1* expression with the HCC patient survival, by analyzing in TCGA HCC cohort data using UCSC Xena database^32^. We stratified the cohort into *DDR1* high and low expression groups. The Kaplan–Meier analysis showed that *DDR1*^*high*^ patient group had lower survival than the *DDR1*^low^ patients (*p* < 0.039) (Fig. 6e). When *PROM1* was used to stratify the cohort, *PROM1*^*high*^ patients had a significantly worse outcome in their survival compared to the *PROM1*^low^ group (*p* < 0.021) (Fig. 6f). When *PROM1* and *DDR1* co-expression was analyzed, 23 patients with high expressions of both *PROM1* and *DDR1* had a worst outcome compared to 69 patients with both genes expressed at the low level (*p* < 0.014) (Fig. 6g). The difference in survival in these patients was greatest (> 1825 days). In summary, *DDR1* is induced in human cirrhosis, HCC, and ICC and inversely associated with the survival of HCC patients. In addition, the expression of *DDR1* is correlated with *PROM1* expression and predicts the survival of HCC patients in this TCGA cohort. These data suggest that *DDR1* expressing PROM1^+^ DRPs and TICs may determine the clinical course of HCC patients.

### Functional significance of DDR1 in TIC-derived tumor development

**Figure 7:**
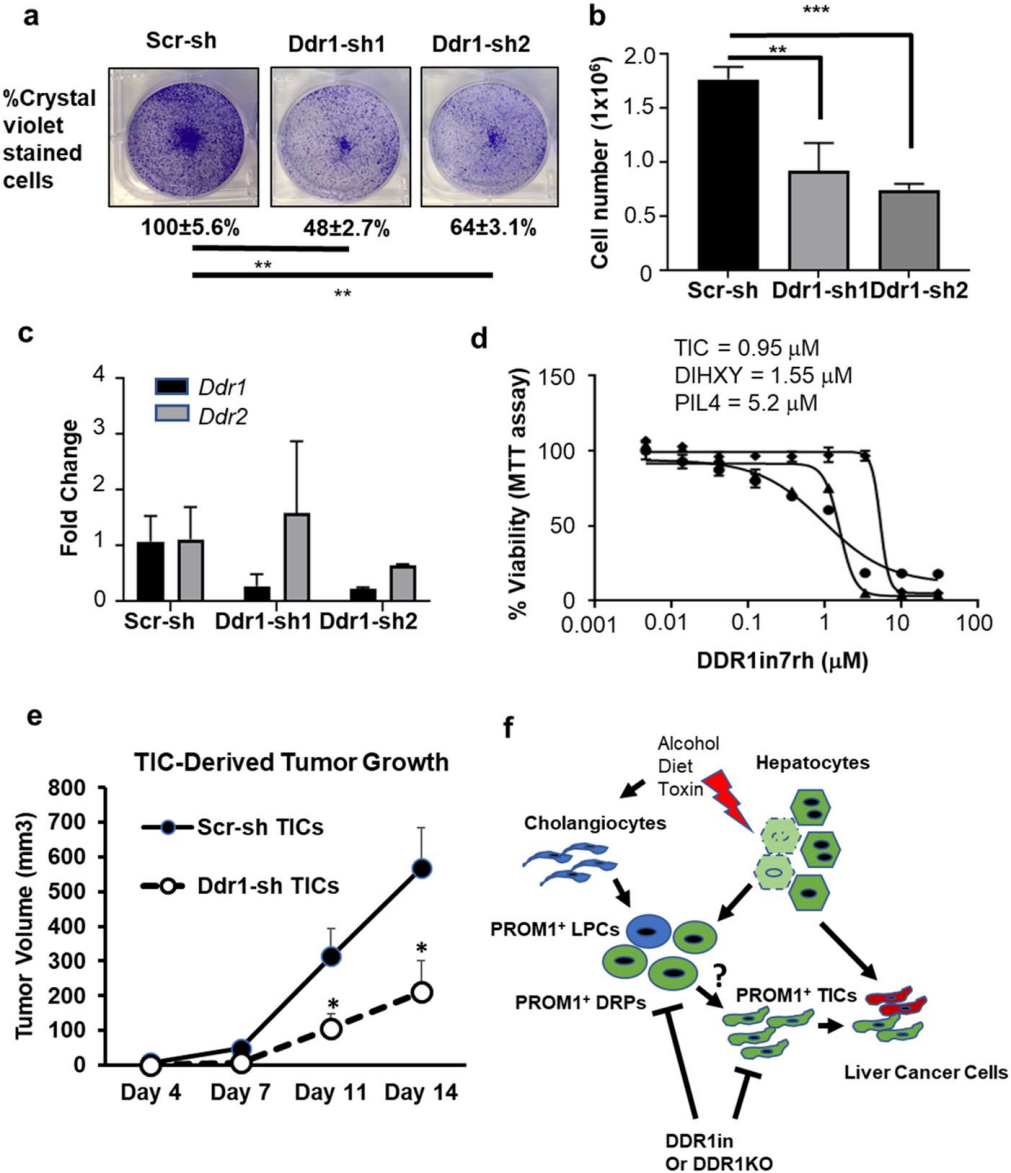
*Ddr1* is required for TIC growth and TIC-derived tumor development in mice. (**a**,**b**) TICs transduced with lentivirus expressing scrambled shRNA (*Scr-sh*), or two *Ddr1*shRNAs (*Ddr1-sh1* and *Ddr1-sh2*) were grown in 6-well plates for 4 days. The cell number was quantitated with crystal violet staining (**a**) and trypan blue cell counting (**b**). Average and SEM were calculated from three experiments. **p < 0.01 and ***p < 0.005 based on One-Way ANOVA with Bonferroni post-test. (**c**) *Ddr1* knockdown in TICs was confirmed by qPCR. (**d**) TICs (circles), DIHXY (triangles) and PIL4 (diamonds) cells were treated with a DDR1 specific inhibitor at a range of concentrations and cell viability was assessed by MTT assay. Each data point represents an average of six replicates. IC_50_ values were calculated using Graphpad Prism. (**e**) TICs transduced with *Scr-sh* or *Ddr1-sh1* were subcutaneously transplanted in nude mice for assessment of tumor development. Each data point is average and SEM of tumor volume from n = 6. *p < 0.05 versus *Scr-sh* TICs. (**f**) The proposed model of the role PROM1^+^ progenitors in liver cancer development. Hepatocyte damage caused by hepatotoxins, alcohol and high fat diet, stimulates proliferation of ductular reaction progenitors (DRPs) including PROM1^+^ liver progenitor cells (LPCs). These PROM1^+^ cells have potential to maintain the DRP lineage and to undergo a transition to tumor cells, possibly via PROM1^+^ TICs. PROM1^+^ DRPs and TICs have high expression of *Ddr1* which drives oncogenesis in the latter cells*.* Inhibition of DDR1 may suppress the contribution of PROM1^+^ cells to liver cancer development.

To determine the functional significance of DDR1 expressed in PROM1^+^ cells in liver oncogenesis, we first measured *Ddr1* expression in TICs, PIL4 liver progenitors and mouse primary hepatocytes. TICs were isolated from a mouse model of alcohol-promoted liver cancer^4^. PIL4 cells were previously isolated from p53-null mouse fed with choline-deficient, ethionine-supplemented diet^35^. TICs have self-renewing and tumor-initiating activities while PIL4 cells lack these properties but mimic DRPs. As expected, albumin is expressed exclusively by primary hepatocytes but not by TICs and PIL4 cells while *Krt19* (CK19) is expressed by PIL4 cells. *Tlr4* which is induced in stem cell-like cells^4^, is expressed moderately by PIL4 cells and most conspicuously by TICs along with *Nanog*, *Sox2* as previously reported (Suppl. Figure 6). A pattern of *Ddr1* expression followed that of *Tlr4*: undetectable in primary hepatocytes, moderately increased in PIL4 cells, and highest expression (~ 40-fold) in TICs. When *Ddr1* but not *Ddr2* was selectively knocked down 80% in TICs by using two separate shRNAs (*Ddr1-sh1* and *Ddr1-sh2*) versus scrambled shRNA (*Scr-sh*) (Fig. 7c), TIC growth was significantly impaired as shown by crystal violet staining and cell count (Fig. 7a,b). Further, the treatment with the DDR1 inhibitor (DDR1in7rh), suppressed the growth of TICs at IC_50_ of 0.95 µM and that of the liver cancer cell line DIHXY at IC_50_ of 1.55 µM while PIL4 cells were four–fivefold less sensitive (Fig. 7d). These results suggested that DDR1 has a functional significance in TIC and liver cancer cell growth. To test this potential oncogenic role of DDR1 in vivo, we performed a xenograft experiment in nude mice transplanted with TICs transduced with *Ddr1-sh1* versus *Scr-sh*. *Ddr1* knockdown in TICs significantly attenuated TIC-derived tumor growth to one third of that with TICs with Scr-sh, supporting the functional role of DDR1 in tumor development (Fig. 7e).

## Discussion

Our study provided several new findings on the origin, fate and functional role of PROM1^+^ cells in alcohol associated liver injury and tumorigenesis. Firstly, using lineage tracing, we showed hepatocytes are in part the origin of PROM1^+^ cells in AH. Secondly, our RNAseq analysis revealed these PROM1^+^ cells have a unique gene expression profile consistent with their characteristics as LPCs/DRPs or TICs/tumor cells. Thirdly, in the DEN-initiated, WAD-promoted liver tumor model, *Prom1*^+^ cells gave rise to DRPs and more importantly tumor cells. Yet, biallelic *Prom1* knock-in mice which lack *Prom1* expression, still developed liver tumors as WT mice, suggesting that PROM1 is a pivotal marker for DRPs/TICs which generate tumor cells while PROM1 itself is not required for tumor development. Fourthly, we have developed a unique method of enriching LPCs and TICs using *Col1a1*-based labeling and FACS. This allowed us to identify two *Prom1*^+^ subpopulations by scRNAseq analysis which we believe represent DRPs and TICs based on their similar yet distinct gene expression profiles. Fifthly, our integrated bioinformatic analysis of RNAseq and scRNAseq data disclosed *Ddr1* as a gene uniquely upregulated in PROM1^+^ cells, and our in vitro and in vivo loss-of-function approaches revealed the oncogenic role of *Ddr1* in TICs. Finally, the clinical relevance and importance of DDR1 was supported by *DDR1* induction in patient HCC and its significant association with low patient survival.

The fate of LPCs has actively been investigated in different mouse models and generated conflicting or mixed results. Lineage tracing studies showed that cholangiocytes defined by KRT19^+^ or SOX9^+^ cells rarely gave rise to hepatocytes^36,37^. In line with this observation, KRT19^+^ or SOX9^+^ cells did not develop HCC in mice, further suggesting that cholangiocyte and hepatocyte lineages may not merge^10,38^. However, recent studies using dual lineage tracing of cholangiocytes and hepatocytes revealed that a significant portion of SOX9^+^ LPCs differentiated into both cholangiocytes and hepatocytes^39,40^. Moreover, KRT19^+^ cholangiocytes became hepatocytes when hepatocyte proliferation was impaired by p21 overexpression or feeding mice with choline deficient ethionine supplemented diet, methionine and choline deficient diet or DDC containing diet^41^. In contrast to these studies which utilized *Sox9*, *Krt19* and *Spp1* as a driver of LPC specific labeling, we used *Prom1-Cre* to determine the fate of LPCs/DRPs and TICs in liver tumor development based on their shared expression of *Prom1*. Our results revealed that PROM1^+^ cells which were derived in part from hepatocytes in AH, gave rise to both DRPs and liver tumor cells in the DEN-WAD mouse model. Because of TICs’ known tumor-initiating activity^4^, labeled TICs were presumed to have generated tumor cells while labeled DRPs must have persisted or expanded in the model. However, we did not observe labeled hepatocytes, suggesting no generation of hepatocytes from PROM1^+^ cells in this model. Our method could not allow selective fate mapping of DRPs versus TICs to clarify their relative contribution to generation of tumor cells. Thus, we cannot rule out a possibility of PROM1^+^ DRPs giving rise to tumor cells either directly or through TICs. From a list of genes shown to be uniquely expressed in *Prom1*^+^ *Afp*^+^ cells versus *Prom1*^+^ *Afp*^–^ or *Prom1*^–^*Afp*^+^ cells, we may be able to devise a method of selective TIC or DRP labeling in future.

Intratumoral heterogeneity presents a unique challenge for understanding of HCC development and therapy resistance. Like other cancers, HCC comprises diverse tumor clones that develop via clonal evolution. HCC in patients with expression of tumor stem cell markers such as *EpCAM*, *PROM1* and *KRT19* tend to be aggressive and chemoresistant. Recent single cell genomics has identified an *EpCAM* + subpopulation with the stemness property with CTSE as an oncogene uniquely induced in this subpopulation^42^. In our study, *DDR1* was identified as a tumor driver gene in *PROM1*^+^ subpopulation relevant to both mouse and patient liver cancer. In particular, patients with combined HCC-ICC having co-overexpression of *DDR1* and *PROM1* had the worst survival, suggesting the contribution of PROM1^+^ TICs to this clinical phenotype. We also identified a group of genes uniquely upregulated in *Prom1*^+^ *Afp*^+^ cells which obviously deserve further attention. Of interest, three of the 4 top genes expressed in this subpopulation (Fig. 4e), are metabolic genes: *Gulo*, beta-catenin responsive gene which encodes the limiting enzyme in the final step in ascorbic acid biosynthesis^43^, *Cyp2c70*, a key enzyme in generation of the murine specific bile acid, a-muricholic acid, or *Elovl2*, an enzyme involved in elongation of long chain w-3 and w-6 fatty acids which was shown to confer stemness of glioma cells^44^. *Prom1*^+^ *Afp*^+^ cells may constitute therapy-resistant TICs in liver cancer^45^ and are a logical and attractive therapeutic target. In fact, in cancer of other organs such as lung, breast, colon, and prostate, DDR1 is already considered as a therapeutic target^28,29,46^. A strategy to selectively direct interventions to key driver genes such as *DDR1* in PROM1^+^ TICs may be desired for the therapy for liver cancer. Finding a molecular link between liver injury and liver carcinogenesis has been a topic of interest. Recently, the DAMP high mobility group box 1 (HMGB1) is shown to underlie hepatocyte-to-ductal metaplasia in liver injury and HCC development in mice^47^. However, the molecular mechanism involved in the potential transition of LPCs to TICs or tumor cells are elusive. Our findings suggest that DDR1 may at least in part facilitate a potential tumorigenic link of PROM1^+^ cells to liver cancer (Fig. 7f). In fact, our study is first to show that DDR1 is essential for TIC growth and TIC-derived tumor formation. Future studies are obviously needed to determine how DDR1 participates in the transition of PROM1^+^ cells to liver tumor cells in vivo. In summary, our study provides the evidence for hepatocyte-origin of PROM1^+^ cells in AH and the functional role of such cells in liver tumorigenesis (Fig. 7f).

## Methods

### Animals and animal models

All animal experiments were performed by the Animal Core of the Southern California Research Center for ALPD and Cirrhosis in accordance to an approved Institutional Animal Care and Use Committee (IACUC) protocol of University of Southern California (USC). C56/BL6, *Prom1*^*C-L*^ (*Prom1*^*tm1(cre/ERT2)Gilb*^) and *Rosa26mTmG (Gt(ROSA)26-Sor*^*tm4(ACTB-tdTomato,-EGFP)*Luo^) were purchased from the Jackson Laboratory and crossed to generate *Prom1*^*C-L*^*; Rosa26mTmG(fl/fl)* mice for the lineage tracing. AAV8/DJ-Ttr-Cre expressing Cre recombinase gene under hepatocyte specific Ttr promoter was purchased from Vector Biolabs and 4 × 10^11^ GC was injected into each mouse via tail vein. *Col1a1Cre;Rosa26mTmG* mice were also generated for FACS-based isolation of *Col1a1* + liver cell population for scRNAseq analysis. For a model of alcoholic hepatitis, the mice were fed 40% of their daily caloric intake as ad libitum intake of Western solid diet containing 1% (w/w) cholesterol and saturated fat and the remaining 60% by intragastric feeding of alcohol-containing liquid diet. They were also superimposed with weekly binge as previously described^14^. For DEN-initiated, WAD-promoted liver tumorigenesis, two-week old male mice were injected with 25 mg/kg of DEN intraperitoneally (Sigma Aldrich, St Louis). For lineage tracing in *Prom1*^*C-L*^*; Rosa26mTmG(fl/fl)* mice, the mice were intraperitoneally injected with two doses of 75 mg/kg tamoxifen (Sigma Aldrich, St Louis) at the 3-day interval at 3 weeks after DEN injection. Western alcohol liquid diet (WAD) (#710362; Dyets Inc.) was introduced to the mice at 5 weeks old by incrementally increasing alcohol concentration in the diet, reaching the maximal concentration of 3.5% (v/v). After 5–6 months of WAD feeding, the mice were euthanized, the livers were carefully excised, weighed and visible tumors were counted. Each tumor length (L) and width (W) were measured by a caliper and the tumor volume was calculated using the formula of (LxW^2^)/2. For TIC xenograft transplantation model, NCr nude mice (Taconic Biosciences) were subcutaneously transplanted with 1 × 10^5^ TICs transduced with shRNA against *Ddr1* or scrambled shRNA, and TIC-derived tumor growth was monitored for 2 weeks by measuring the tumor length and width for assessment of tumor volume as described above.

### Cell culture

TICs were provided by the Integrative Liver Cell Core (ILCC) of USC and cultured in 1:1 mixture of Dulbecco's modified Eagle medium (DMEM) and Nutrient Mixture F‐12 media (Life Technologies, Camarillo, CA) with 10% fetal bovine serum (FBS), 20 ng/mL murine endothelial growth factor (EGF; Peprotech, Rocky Hill, NJ), Embryomax nucleosides (MilliporeSigma, Burlington, MA), 100 nM dexamethasone (Sigma–Aldrich, St. Louis, MO), and antibiotic‐antimycotic (Life Technologies) as described before^4,45^. DIHXY cells were cultured in DMEM medium with 10% fetal bovine serum (FBS) and antibiotic‐antimycotic (Life Technologies). PIL-4 cells were obtained from Dr. George Yeoh of University of Western Australia^35^ and cultured in Williams’ E medium supplemented with 10% FBS, EGF (Peprotech), IGF-II (Peprotech) and human Insulin (Sigma–Aldrich, St. Louis). The DDR1 inhibitor 7rh was purchased from Sigma–Aldrich for the treatment of TICs in culture. MTT assay and crystal violet assay were performed as described before^45^.

### Alcoholic hepatitis patient and healthy liver tissues

AH patient and control human livers were obtained from the Clinical Resources for Alcoholic Hepatitis Investigators (1R24AA025017) of Johns Hopkins University as previously described^24^. The patient samples were obtained with informed consent in accordance with Johns Hopkins Hospital Institutional Review Board as described before^41^. These tissues were excised from explanted livers in patients with severe AH during liver transplantation, or wedge biopsies from the donor livers (normal control). Suppl. Table 1 summarizes basic clinical information of 4 AH patients and 4 healthy donors from which the samples we analyzed were collected.

### Immunohistochemistry and immunofluorescence staining

The liver slices were fixed in 4% paraformaldehyde (PFA) at 4^◦^C overnight and transferred to 70% ethanol on the next day. The fixed tissues were paraffin-embedded, and paraffin sections were stained with hematoxylin & eosin (H&E). For immunofluorescence staining (IF), the liver slices were fixed in 4% PFA at 4^◦^C for 2 h, incubated in10% sucrose in PBS for 2 h and 30% sucrose in PBS for overnight. The tissues were then frozen in the freezing medium and sectioned with a Cryostat (CM1900; Leica, Buffalo Grove, IL) to prepare 7 µm thickness cryosections. The sections were air-dried overnight, treated with 3% H_2_O_2_ in methanol and permeabilized with proteinase K prior to blocking with the solution containing 5% donkey serum and 0.2% bovine serum albumin for 30 min. The sections were then incubated with primary antibodies against Green Fluorescent Protein (GFP; 400-fold dilution, Rockland, Limerick, PA) or KRT19 (TROMAIII; 50-fold dilution, Developmental Studies Hybridoma Bank, Iowa City, IA), subsequently incubated with secondary antibodies: Donkey anti-Goat conjugated with AlexaFluor488 and AlexaFluor 550 (400-fold dilutions, Thermo Fisher Scientific) for 30 min. The sections were counterstained with DAPI. Images were captured with a Nikon 90i microscope and DS-Fi1 digital camera (Nikon, Melville, NY). For IF shown in Fig. 3b, the liver section was stained for HNF4α (50-fold dilution, #6556, Santa Cruz Biotechnology), KI67 (50-fold dilution, #652,402, Biolegend), SOX9 (500-fold dilution, #AB5535, Millipore) as previously described before^48^. Images were captured with a confocal microscope.

### FACS isolation

Isolation of PROM1^+^ cells was performed by the ILCC of USC. The liver was perfused with EMEM medium, minced on a 10 cm dish and resuspended in collagenase IV containing digestion medium (25 mg of collagenase type IV (C5138-Sigma Aldrich) in 50 mL of serum free DMEM-F12, pH 7.35–7.4). Hepatocytes were removed by low speed centrifugation. CD45^+^ cells were depleted with CD45 microbeads (Miltenyi Biotec). The cells were blocked with FcR blocking reagent and stained with the corresponding antibodies. CD133-APC, CD49f.-PE and CD45-efluor450 (eBioscience). The single controls and Fluorescent Minus One (FMO) were added in each experiment to gate the positive population. PI was added to separate live cells from dead cells. The cells were sorted in MoFlo Cell Sorter (Beckman Coulter). Isolation of *Col1a1* expressing cells was carried out by using the protocol previously described^49^. A non-parenchymal liver cell fraction from *Col1a1-Cre;Rosa26mTmG* mice was collected by pronase-collagenase perfusion and gradient ultracentrifugation. Gating for UV and GFP positivity were set by primary hepatic stellate cells isolated from wild type mouse and *Col1a1Cre;Rosa26mTmG* mouse, respectively, with wild type liver myofibroblasts as a UV and GFP negative cells. UV^–^GFP^+^ fraction was collected for scRNAseq analysis.

### RNA sequencing

The cells were centrifuged and total RNA was isolated with PicoPure RNA isolation kit (Thermo Fisher Scientific). Prior to library construction RNA integrity was verified by Experion analysis (Bio-Rad). PolyA selection was carried out using Illumina Truseq V2 polyA beads. Sequencing was performed on a NextSeq 500 using V2 chemistry. RNA-seq data was analyzed using the RNA-seq workflow in Partek Flow software (v3. Partek Inc., St. Louis, MO, USA). Briefly, the raw sequencing reads were first trimmed based on the quality score (Phred QC >  = 20, min read length = 25 nt) before mapped to mouse genome build mm10 using Tophat version 2.0.8 with default parameter settings and using Gencode M3 annotation as guidance. Gencode M3 was then used to quantify the aligned reads to genes using Partek E/M method. The gene level aligned read counts were normalized for all samples using Upper Quartile normalization before subjected to differential expression analysis using Partek Gene Specific Analysis method (genes with fewer than 10 aligned reads in any sample among a data set were excluded). The differentially expressed gene (DEG) lists were generated using the cutoff of FDR < 0.05 and fold changes greater than 2 either direction.

### Single-cell RNA sequencing

Single cell was prepared using the 10 × Single Gene Expression v3 with the 10 × Chromium technology following manufacturer protocol as described before^50,51^ (cat# 1000154 and 1000074, 10 × Genomics). The library was prepared immediately after cell sorting with the target cell number of 10,000. The library was validated on the Agilent TapeStation (Agilent Technologies, Palo Alto, CA, USA), and quantified by using Qubit 2.0 Fluorometer (Invitrogen, Carlsbad, CA) as well as by quantitative PCR (KAPA Biosystems, Wilmington, MA, USA). The sequencing libraries were clustered on 8 single lanes of a flowcell. After clustering, the flowcell was loaded on the Illumina HiSeq instrument according to manufacturer’s instructions. The samples were sequenced using a 2 × 150 Paired End (PE) configuration. Raw sequence data (.bcl files) generated from Illumina HiSeq was converted into fastq files and de-multiplexed using the 10 ×  Genomics’ cellranger version 3.1.0 by the Cell Ranger mkfastq command with default parameters. Subsequently, UMI and cell barcode de-convolution and mapping to the mm10 reference genome was performed with 10 × Genomics’ Cell Ranger software package (version 3.1.0) to generate the final digital gene expression matrices and cloupe files using the Cell Ranger count command with default parameters. The quality of data is provided as a barcode rank graph (Suppl. Figure 4). Of targeted 10,000 cells, 8578 cells were subjected for sequencing. After normalization, 38,901 mean reads per cell and 2776 median genes per cell were detected. Data analysis was performed with 10 × Genomics’ Loupe Browser software. Raw data files were uploaded to GEO database with accession number GSE152748.

### Quantitative PCR

RNA extraction was performed using Quick RNA miniprep kit (Zymo Research). Tissues were homogenized in RNA lysis buffer. RNA was extracted according to the manufacturer’s protocol. 500–1000 ng of RNA was used as input to synthesize cDNA using high-capacity cDNA reverse transcription kit (ThermoFisher Scientific). Q-PCR analysis was performed using the primers shown in Suppl. Table 1 and Sybr Green reagents (ThermoFisher Scientific) in Viia7 instrument (Applied Biosystem).

### Lentiviral mediated Ddr1 knockdown

Ddr1 mission shRNA were purchased from Sigma Aldrich. Two shRNAs with the following sequences were chosen: Ddr1shRNA1 (CCGGGATTCCACTTACGATGGA-TATCTCGAGATATCCATCGTAAGTGGAATCTTTTT) and Ddr1shRNA2 (CCGGGC-CACGCTGAACTTTGTGCATCTCGAGATGCACAAAGTTCAGCGTGGCTTTTT). The shRNAs were packaged into lentivirus with psPAX2 and pMD2.G in 293FT cell line. The lentiviruses were purified using speedy lentivirus purification kit (LV999, Applied Biological Materials Inc., Canada) and titrated using qPCR lentivirus titration kit (LV900, Applied Biological Materials Inc., Canada). The cells were infected with 10 MOI of virus and cultured in 10 µM of puromycin (Invitrogen) after 48 h of virus infection.

### Bioinformatic analysis

Bioinformatic analysis was performed by using University of California Santa Cruz (UCSC) Xena program. TCGA liver cancer cohort was selected with the filters: primary tumors and hepatocellular carcinoma as primary disease. *DDR1* and *PROM1* expression were extracted from the gene expression generated by IlluminaHiseq.

### Data analysis

RNAseq data was analyzed by Partek Flow (St. Louis, MO) and performed by USC libraries Bioinformatics Services. Pathway analysis was performed by Ingenuity Pathway Analysis (Qiagen, Redwood City, CA). Statistical Analysis was performed by Graphpad Prism (San Diego, CA) and Microsoft Excel.

## Supplementary information

Supplementary Information.

Supplementary Table S2.

Supplementary Table S3.

Supplementary Table S4.

Supplementary Table S5.
