# Supplementary material for: Fate and functional roles of Prominin 1+ cells in liver injury and cancer
Source: Sci Rep. 2020 Nov 10;10:19412. doi: 10.1038/s41598-020-76458-8 (PMC7656457; doi:10.1038/s41598-020-76458-8)
Supplement: Supplementary file 1 — Supplementary Information. [file 41598_2020_76458_MOESM1_ESM.docx]

**Supplementary Information**

**Title: Fate and Functional Roles of Prominin 1^+^ Cells in Liver Injury and Cancer**

**Author Information**:

Raymond Wu, Stephanie Pan, Yibu Chen, Yasuhiro Nakano, Meng Li, Steven Balog, Hidekazu Tsukamoto

**Suppl. Table1:** The primer sequences used in qPCR analyses were obtained from Harvard Primers Bank.

| Mouse Primers | Forward (5’ to 3’) | Reverse (5’ to 3’) |
| --- | --- | --- |
| *Spint1* | GTCGGCGTATGGCTCCTTT | GCTTCGGTGTCCAGCACAA |
| *Ddr1* | ATGCTGACATGAAGGGACATTT | GGTGTAGCCTACGAAGGTCCA |
| *Ddr2* | ATCACAGCCTCAAGTCAGTGG | TTCAGGTCATCGGGTTGCAC |
| *Lamc2* | CAGACACGGGAGATTGCTACT | CCACGTTCCCCAAAGGGAT |
| *Lyve1* | CAGCACACTAGCCTGGTGTTA | CGCCCATGATTCTGCATGTAGA |
| *Klf2* | CTCAGCGAGCCTATCTTGCC | CACGTTGTTTAGGTCCTCATCC |
| *Ehf* | CAGGAGTTCGACATTAGCGGA | TCTACTGTGCTACCATAGCTGG |
| *Gfra1* | CACTCCTGGATTTGCTGATGT | AGTGTGCGGTACTTGGTGC |
| *Kdr* | TTTGGCAAATACAACCCTTCAGA | GCAGAAGATACTGTCACCACC |
| *Gadd45g* | GGGAAAGCACTGCACGAACT | AGCACGCAAAAGGTCACATTG |
| *Clec4g* | ACTGGTGAATACAACAAGCTGG | ACTGGACAGTAGGGTGCTCAG |
| *Ehd3* | AGTTGGCTGGGTAACGATGAT | GTGGTCTTGCCGGTAGAGT |
| *Ptprb* | TCAAGGCAGGACAGTACCC | TGTATTTCTCCCATTCGCCTAGA |
| *Pkhd1* | GGGAGGTCGATGGTGCATAAG | GATGTCCGTTCTTCCCCCAAG |
| *Cftr* | CTGGACCACACCAATTTTGAGG | GCGTGGATAAGCTGGGGAT |
| *Lamc3* | CGGAGCCCTGCATCACAAA | AGCAAGGTCGTCCTCAAAGC |
| *Alb* | TGAGTGAGCATGTTACCAA | AGAGCAGAGAAGCATGGC |
| *Tlr4* | GGCAACTTGGACCTGAGGAG | CATGGGCTCTCGGTCCATAG |
| *Nanog* | AGGGTCTGCTACTGAGATGCTCTG | CAACCACTGGTTTTTCTGCCACCG |
| *Sox2* | GGCAGCTACAGCATGATGCAGGAGC | CTGGTCATGGAGTTGTACTGCAGG |
| *Col1a1* |  |  |
| *36B4* | AGATTCGGGATATGCTTGTTGGC | TCGGGTCCTAGACCAGTGTTC |

| Human Primers | Forward (5’ to 3’) | Reverse (5’ to 3’) |
| --- | --- | --- |
| *DDR1* | CCGACTGGTTCGCTTCTACC | CGGTGTAAGACAGGAGTCCATC |
| *DDR2* | GCTATATGCCGCTATCCTCTGG | ACTCTGACCACTGACTGGAAG |
| *36B4* | CAGATTGGCTACCCAACTGTT | GGAAGGTGTAATCCGTCTCCAC |

**Suppl. Table 2.** Summary of AH patients (A1-4) and healthy donors (C1-4)

|  | Age | Sex | Decompensation | Sepsis | Maddrey’s DF | PT (sec) | Bilirubin (mg/dl) |
| --- | --- | --- | --- | --- | --- | --- | --- |
| A1 | 32 | M | Yes | No | 77.4 | 18.4 | 38.8 |
| A2 | 34 | F | Yes | No | 55.3 | 16.0 | 27.7 |
| A4 | 48 | M | Yes | No | 187.4 | 40.3 | 48.0 |
| A5 | 41 | M | Yes | No | 147.0 | 34.7 | 33.4 |
| C1 | 55 | M | n/a | No | n/a | n/a | n/a |
| C2 | 61 | F | n/a | No | n/a | n/a | n/a |
| C3 | 27 | F | n/a | No | n/a | n/a | n/a |
| C4 | 32 | M | n/a | No | n/a | n/a | n/a |

DF, Discriminant Function; PT, prothrombin time.

From Clinical Resource for Alcoholic Hepatitis Investigations at Johns Hopkins University (R24AA025017)

**Suppl. Table 3:** RNAseq DEG list for PROM1+ cells from mouse AH liver – Attached as an Excel file.

**Suppl. Table 4:** ScRNAseq gene profiles of *Prom1+Afp+* cells from DEN-WAD tumors – Attached as an Excel file.

**Suppl. Table 5**: ScRNAseq gene profiles of *Prom1^+^Afp^-^* cells from DEN-WAD tumors – Attached as an Excel file.

**Suppl. Table 6**: ScRNAseq gene profiles of *Prom1^-^Afp^+^* cells from DEN-WAD tumors – Attached as an Excel file.


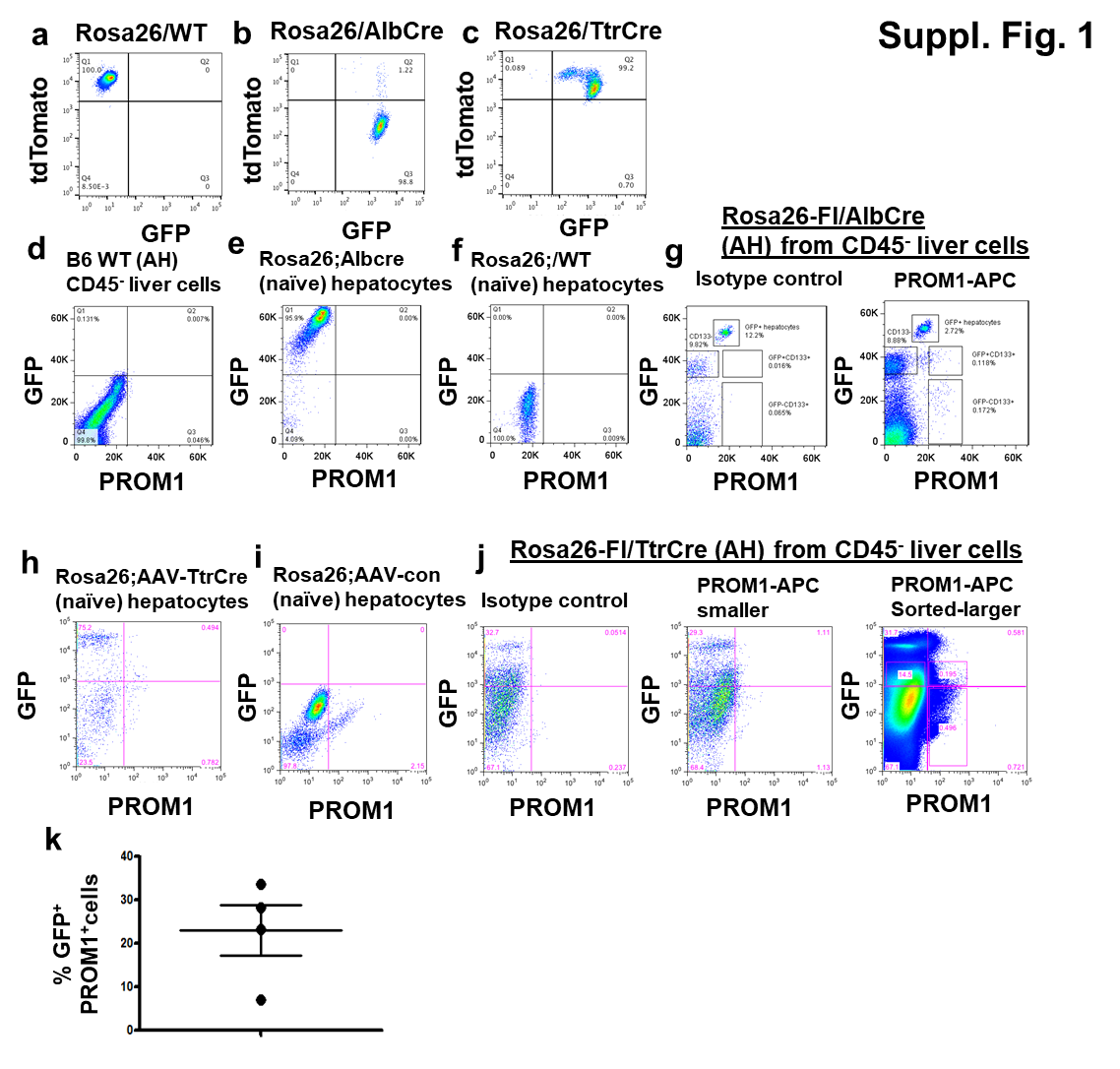


**Suppl. Fig. 1**: Hepatocytes-derived PROM1^+^ cells in mouse model of alcoholic hepatitis (AH). a-c) FACS isolation gated by tdTomato and GFP of hepatocytes isolated from *WT;Rosa26mTmG*, *Alb-Cre;Rosa26mTmG* and *Rosa26mTmG* injected with AAV-TtrCre virus without any challenge. For c), hepatocytes were isolated one month after AAV injection. d) CD45^-^ liver cells sorted with GFP and PROM1-APC gating from the liver of C57/BL6 mice treated with AH protocol but without anti-PROM1-APC antibody. e and f) Hepatocytes isolated from naive *Alb-Cre;Rosa26mTmG*  and *WT;Rosa26mTmG* mice for GFP gating. g) *Alb-Cre;Rosa26mTmG* mice were challenged with AH protocol. CD45^-^ liver cells were stained with anti-PROM1-APC antibody or isotype control for FACS. h) GFP labeling of hepatocytes isolated from *Rosa26mTmG* injected with AAV-TtrCre vs. i) AAV control virus. j) *AAV-TtrCre;Rosa26mTmG* mice were challenged with AH protocol. CD45^-^ liver cells were stained with anti-PROM1-APC antibodies for FACS. k) The percentage of GFP^+^PROM1^+^ cells to total PROM1^+^ cells detected by FACS from 4 AH mice.


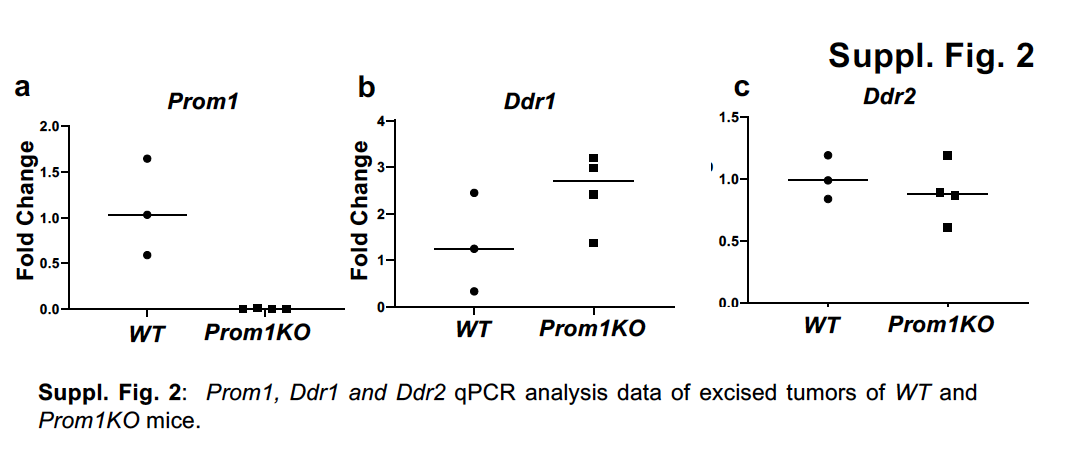


**Suppl. Fig. 3**


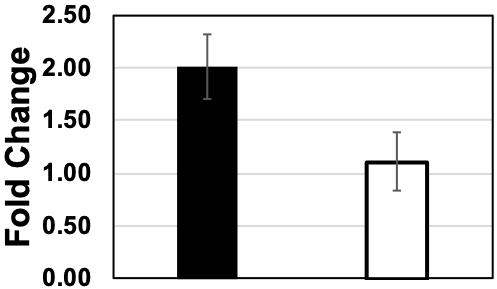


**PROM1^+^**

**PROM1^-^**

***Col1a1***

*****

**TIC**

**PIL4**

**Hep**

***Col1a1***


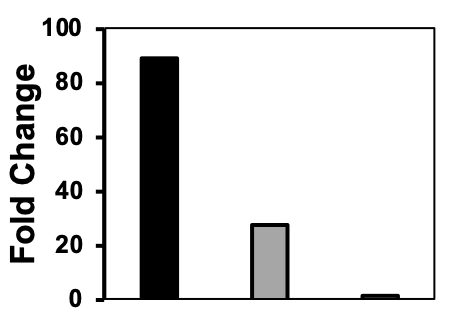


**Suppl. Fig. 3**: **A. q**PCR analysis of *Col1a1* mRNA in mouse primary hepatocytes (Hep), liver progenitor cells (PIL4), and mouse liver tumor initiating cells (TIC). **B.** qPCR analysis of *Col1a1* mRNA in PROM1^+^ and PROM1^-^ cells isolated by magnetic activated cell sorting (MACS) using anti-PROM1 antibody beads from a non-hepatocyte fraction of tumor bearing livers from DEN-WAD mice. RNA was immediately extracted after isolation. The average and SEM were calculated from the values of three independent extractions. *p<0.05, student t-test.


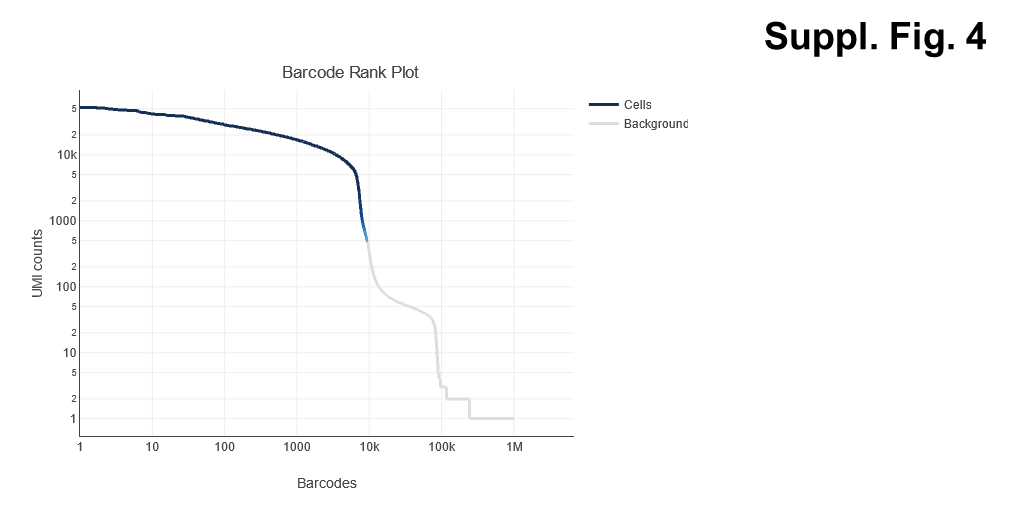


**Suppl. Fig. 4**: Barcode Rank Plot of the scRNAseq of p5 fraction of DEN-WAD liver. The y-axis represents Unique Molecular Identifier (UMI) counts mapped to each barcode. The sharp decrease in UMI indicates a good separation between the cell-associated barcodes and barcodes associated with empty partitions. More information can be obtained from <https://support.10xgenomics.com/single-cell-gene-expression/software/pipelines/latest/output/summary>.


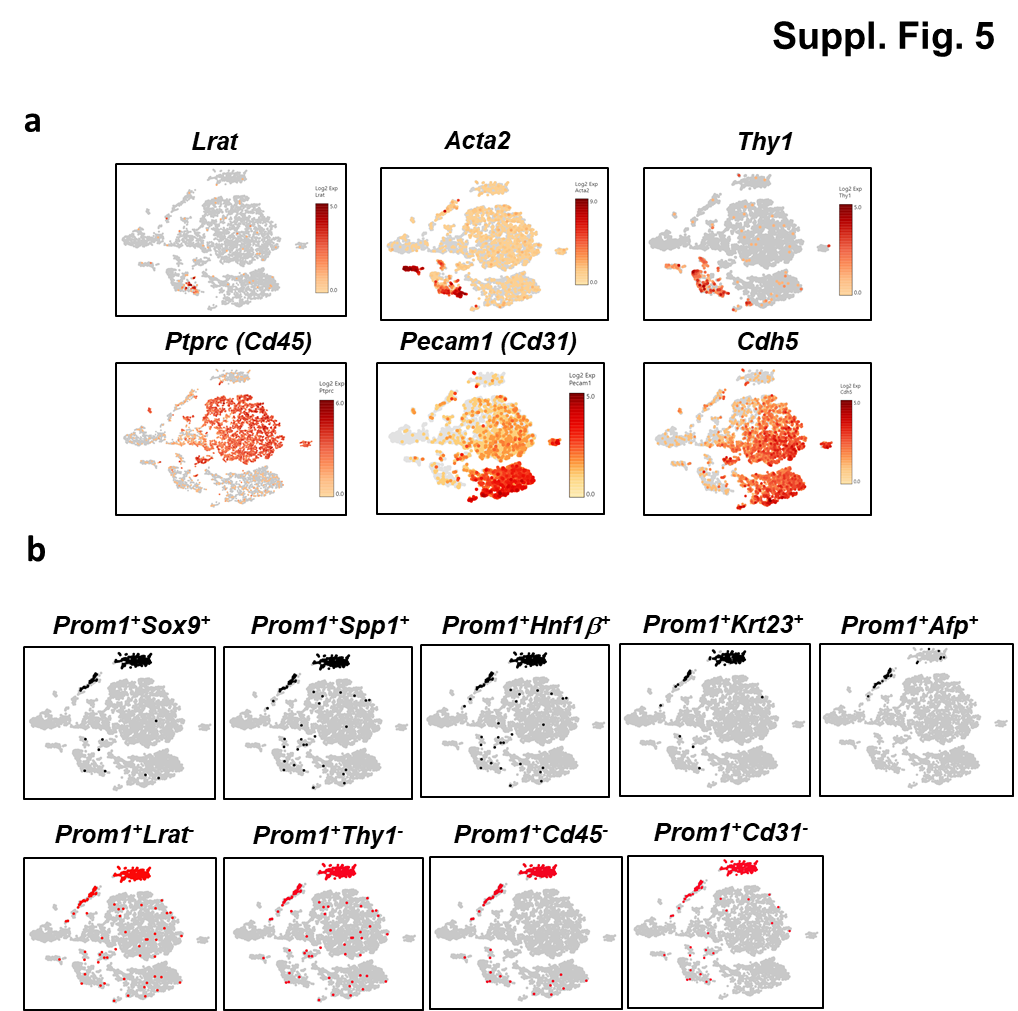


**Suppl. Fig. 5**: a. t-SNE plots of known markers of hepatic stellate cells (*Lrat*), smooth muscle cells (*Acta2*), portal fibroblasts (*Thy1*), hematopoietic cells (*Ptprc/Cd45*), and endothelial cells (*Pecam1/Cd31*, and *Cdh5*) using the gene/feature expression filter. The scale bars represent log2 transformed expression level. b. Co-expression analysis revealing *Prom1+ cells co-*expressing known marker genes of DRPs (*Soz9, Spp1, Hnf1b, Krt23*) and for tumor cells (*Afp*) shown in black dots. Most of these *Prom1+* cells do not co-express hepatic stellate cells (*Lrat*), portal fibroblasts (*Thy1*), hematopoietic cells (*Cd45*) and endothelial cells (*Cd31*) as shown in lower panels.


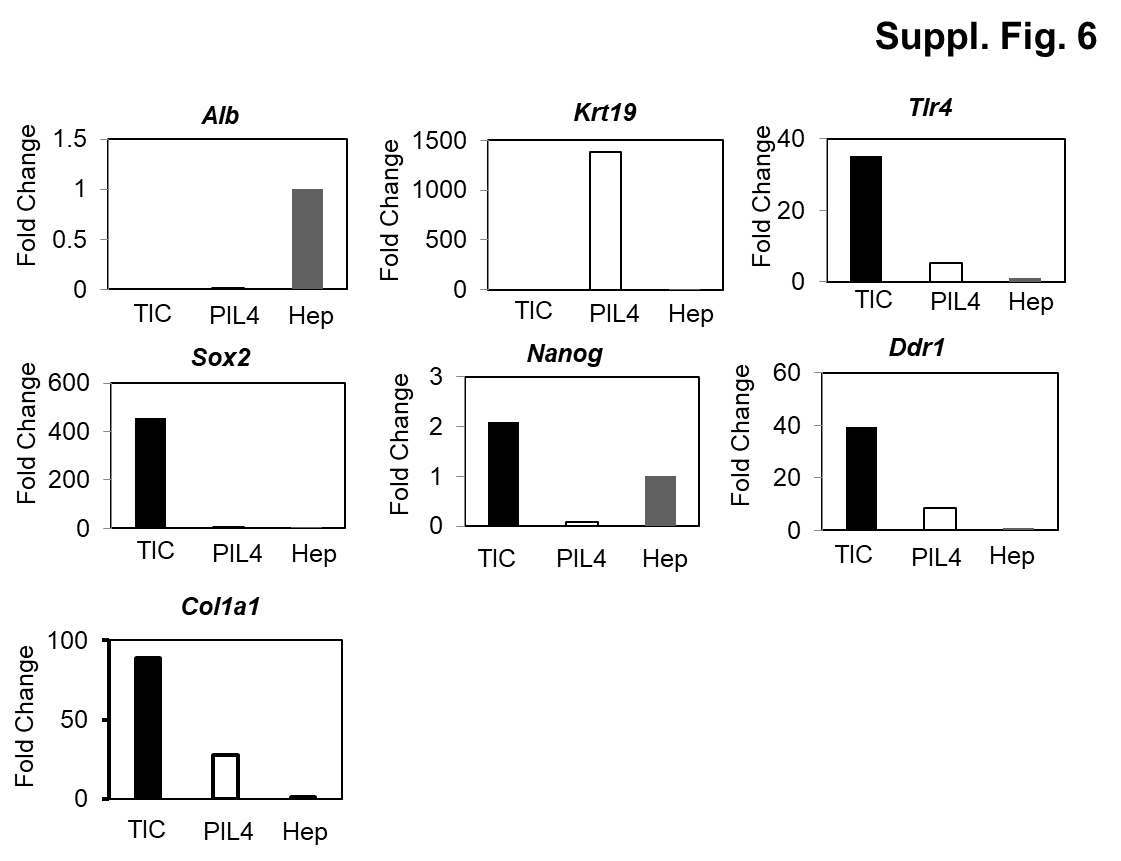


**Suppl. Fig. 6**: qPCR data of hepatocyte (*Alb*), progenitor (*Krt19*), TIC (*Tlr4, Sox2, Nanog*) marker genes along with *Ddr1* and *Col1a1* in TICs, PIL4 and mouse primary hepatocytes.
